# The Effectiveness of Adjuvant Attangaogam (Athanam) Yoga Asana-Pranayamam Practices With Regard to Biochemical, Inflammatory, and Hematological Markers Among COVID-19 Patients at a Tertiary Care Hospital in Southern Tamilnadu

**DOI:** 10.7759/cureus.38727

**Published:** 2023-05-08

**Authors:** Suganthy K, Lakshmiprabha S, Virgin Joena M, Vishnu shankar Raja MR, Raj Kishore Mahato, Hariharan A, Mamatha T Shenoy, Shanmugapriya V, Pradipta Kumar Mohanty

**Affiliations:** 1 Biochemistry, Velammal Medical College Hospital and Research Institute, Madurai, IND; 2 Biochemistry, Vinayaka Missions Medical College & Hospitals, Karaikal, IND; 3 Medicine, Velammal Medical College Hospital and Research Institute, Madurai, IND; 4 Yoga and Naturopathy, Attangaoga Peedam, Tirunelveli, IND; 5 Pharmacology, Velammal Medical College Hospital and Research Institute, Madurai, IND

**Keywords:** covid-19, attangaogam, yoga, pranayamam, biochemical markers, inflammatory markers

## Abstract

Background and objective

Coronavirus disease 2019 (COVID-19), caused by severe acute respiratory syndrome coronavirus 2 (SARS-CoV-2), is a highly contagious infectious disease that has affected many countries globally. Attangaogam, known in Sanskrit as "Ashtanga yoga", is a practice associated with the spiritual and cultural heritage of India whose origins can be traced back to the very dawn of civilization; the practice of yoga promotes health, healing, and longevity. This study aimed to analyze the effects of Attangaogam (Athanam) yoga asana-Pranayamam practice on biochemical, inflammatory, and hematological markers in the management of COVID-19.

Materials and methods

A prospective observational study was conducted from August 2021 to February 2022 among hospitalized adult patients of both sexes who consented to participate and tested positive for COVID-19 reverse transcription-polymerase chain reaction (RT-PCR). Convenience sampling was employed and the study was approved by Institutional Ethics Committee (VMCIEC/74/2021). Clinical details, inflammatory markers, D-dimer, lactate dehydrogenase (LDH), ferritin, procalcitonin (PCT), interleukin 6 (IL-6), and complete blood count (CBC) were analyzed for all the volunteering patients on admission and before commencing yoga-pranayamam practices. Also, the parameters were recorded after practicing the scheduled protocol: on the day of discharge, and after the first and third months of discharge. Microsoft Excel 2013 was used for statistical analysis.

Results

Of the 76 patients, 32 were followed up regularly; the mean age of the cohort was 50.6 ± 4.95 years, and 62% were males. All the patients attained normal oxygen saturation and got discharged in 7-14 days. The comparison of clinical, hematological, inflammatory, and biochemical investigations between pre- and post-Attangaogam yoga-Pranayamam practice sessions showed statistically significant differences and the patients attained normal levels for all variables within three months except for serum albumin.

Conclusion

Based on our findings, the practice of Attangaogam yoga-Pranayamam contributed to the successful treatment of COVID-19 with the early restoration of protracted hypermetabolic and hyperinflammatory markers to normal status. The evidence related to biomarkers revealed that the patients attained metabolic normalcy of cell health with the aid of personalized physical rehabilitation counteracting inﬂammation and promoting tissue repair thanks to holistic natural and innate immunity provided by Attangaogam yoga-pranayamam practices.

## Introduction

Coronavirus disease 2019 (COVID-19) is a highly contagious disease that has wreaked havoc and severely affected individuals and healthcare systems across the world. India was among the most severely affected countries during the COVID-19 pandemic, with over 458,000 deaths reported as of November 1, 2021 [1,2]. The widespread quest for an effective cure for the condition has led people to adopt yoga as a method for treating this serious healthcare concern. The need of the hour is crafting a symbiotic relationship between yoga and modern science. Attangaogam, known in Sanskrit as "Ashtanga yoga", and now called "yoga asana" or Athanam in Tamil [3,4], is one of the 64 sacred arts given as a precious gift to the world by the ancient Tamil Siddhars who were hailed for their true wisdom. The Attangaogam Peedam has adapted this ancient art for the benefit of modern scientific minds by reframing the traditional purity from a modern perspective. Attangaogam comprises eight limbs: Yamam, Niyamam, Athanam, Pranayamam, Prathyaharam, Tharanai, Dhyanam, and Samadhi [3]. Attangaogam is a glorious practice representing the spiritual and cultural heritage of India, dating back thousands of years to the time of great ancient rishis; it promotes health, healing, and longevity. The therapeutic dimension of Attangaogam has been investigated in hundreds of diseases, as evidenced in the bibliography of research compiled by Timothy Mcall [5]. It fosters the restoration of normalcy in all systems of the human body with a special focus on the physical-psycho-neuro-immuno-endocrine axes [4].

These yoga techniques are ideal for the preventive, promotive, curative, as well as rehabilitative aspects of healthcare. It can be an adjunct to modern medical treatment as new breathing techniques can help to improve lung capacity, volume, and function as well as aid in promoting the recovery process in diseases such as flu, common cold, acute respiratory distress syndrome, and COVID-19. They help in early recovery, decrease inflammatory markers, and prevent complications of COVID-19 by improving oxygenation and cardiorespiratory functions and mitigating psychosomatic stress [6,7,8].

In this study, we aimed to assess the effectiveness of Attangaogam yoga asana-Pranayamam practices as a supplementary therapeutic intervention in the management of COVID-19, especially with regard to biochemical, inflammatory, and hematological markers.

## Materials and methods

Study design

This was a prospective observational study conducted after obtaining approval from the Institutional Ethics Committee. The data were collected from patients admitted during the second wave of the COVID-19 pandemic at the COVID isolation ward of a tertiary care hospital between August 2021 and February 2022.

Ethical consideration

Ethical approval was obtained from the Institutional Ethics Committee (ethics code: VMCIEC/74/2021). All participants were informed of the purpose of the study and they signed a written informed consent form. They were informed that they were free to leave the study at any time. All methods were performed in accordance with the tenets of the Declaration of Helsinki.

Study size and sampling

Convenience sampling was employed, and the sample size was calculated using the formula n = Z^2^ X (σ^2^/e^2^) = 1.96^2 ^× (17.7^2^/8.0^2^), where n is the minimum required sample size, Z = 1.96 at 95% confidence interval (CI), e is the margin of error (8.00), and σ is the standard deviation based on a previous study [9].

Study sample

The study initially enrolled 76 patients presenting with COVID-19 infection; 60 participants who volunteered were followed up based on inclusion and exclusion criteria. The data of 32 volunteered COVID-19 patients who regularly attended yoga-Pranayamam practices as per the scheduled protocol and followed up after the first and third months were used for the final analysis.

Inclusion Criteria

All adult patients aged >18 years of both sexes, who provided informed consent, tested positive for COVID-19 reverse transcription-polymerase chain reaction (RT-PCR), who were hospitalized and diagnosed to have mild to moderate symptoms as per the guidelines of the Ministry of Health and Family Welfare (MoHFW), India [9] and were willing to practice yoga asanas (Athanam) and Pranayamam were deemed eligible for inclusion. Also, all potential participants were required to have a smartphone and internet facility and be able to use it by themselves or with the help of family members and to continue the practice sessions online at home.

Exclusion Criteria

COVID-19 patients who did not consent to participate, those with severe symptoms or in the critical category as per the guidelines of MoHFW [9], patients in the ICU or on mechanical ventilation, patients unable to perform yoga or Pranayamam, patients with known psychiatric illnesses, chronic obstructive pulmonary disease (COPD), severe hypertension, malignancy, and pregnant/lactating females were excluded.

The practice and timing schedule of Attangagoga (asanas)-Pranayamam is presented in Table 1.

**Table 1:** Attangagoga (asanas)-Pranayamam practice and timing schedule

Asanas-Pranayamam	Practice rounds: duration		Timing of practice
Golden yoga asanas: 21			
Pujangasanam (Cobra Pose)	1 round: 30 minutes		6 AM-7 AM, 5 PM-6 PM
Muyalasanam (Rabbit Pose)			
Usartasanam (Camel Pose)			
Dhanurasanam (Bow Pose)			
Artha Sirasasanam (Handstand Pose)			
Arthasalaba Asanam (Half Locust Pose)			
Kutha Patha Asanam (Butterfly Pose)			
Vajrasanam (Thunderbolt Pose)			
Sidhapadmasanam (Half Lotus Pose)			
Janu Siras Asanam (Head-to-Knee Pose)			
Padmasanam (Lotus Pose)			
Nindra Patha Asanam (Crane Pose)			
Veera Pathra Asanam (Warrior Pose)			
Utkat Asanam (Chair Pose)			
Thirikona Asanam (Triangle Pose)			
Pirai Asanam (Crescent Pose)			
Macha Asanam (Fish Pose)			
Uthana Patha Asanam (Raised Leg Pose)			
Bavana Muktha Asanam (Wind Liberating Pose)			
Sarvanga Asanam (Shoulder Stand Pose)			
Yoga Nithra/Shanthi Asanam (Sleep Pose)			
Pranayamam: 9			
Nadi Suthi 1, 2, 3 (nostril breathing and regulating technique)	5 rounds: 15 minutes	6 AM-7 AM, 11 AM-12 PM, 4 PM-5 PM, 6 PM-7 PM, 8 PM-9 PM	
Surya-Chandra Nadi (Sun-Moon nostril breathing)			
Kabalapathi (skull-lungs cleansing)			
Seethkali (cooling breathe)			
Seethkadi (sipping Breathe)			
Ujjai (ocean breath)			
Brahmari Pranayama (bee breath)			
After-discharge (online-monitored)			
Golden yoga asanas: 21	1 round: 30 minutes		6 AM-7 AM
Pranayamam	5 rounds: 15 minutes		1-3 times per day

This protocol of 21 asanas and nine pranayamam has been adapted and validated based on various research works [5]. A certified yoga master trained the patients at the bedside in the practice of Attangagoga (asanas)-Pranayamam and supervised the sessions by direct observation in the wards and online after discharge for a duration of three months. The schedule was approved by the Institute Research Advisory Committee and is available in videos and print (pdf) on the website http://www.attangaogam.com; book: http://www.attangaogam.com/Attangaogam.pdf. The link was shared with all the participants.

Asana-Pranayama schedule by direct observation was conducted during 7-10 days of hospital admissions. The patients were reviewed with post-COVID health check-up packages after one and three months of practice. All enrolled patients were daily monitored online on the Zoom platform for one month by the yoga center, and then weekly to confirm their compliance with the regular practice schedule. Also, a WhatsApp group including all the investigators and participants was formed for communicating and sharing supportive links. A daily attendance record was maintained. All the participants were closely monitored by the yoga instructors to ensure adherence to the protocol throughout the study duration.

Study outcome parameters: vital signs and biochemical, hematological, and inflammatory markers

At the time of admission, the COVID-19 Reporting and Data System (CO-RADS) score classification guidelines were used for the categorization of patients into groups based on the likelihood of a patient having confirmed COVID-19 with lung involvement. CO-RADS consists of seven categories. Categories 1-6 represent a progressively increasing risk for COVID-19, ranging from very low risk (CO-RADS 1) to proven infection by a positive RT-PCR assay (CO-RADS 6). Real-time RT-PCR (rRT-PCR) was done [9]. The routine clinical chemistry tests were performed using Toshiba 120FR fully automated system (Canon Medical Systems Corporation, Tochigi, Japan) and the complete blood count (CBC) was performed using Beckman Coulter LH 750 (Beckman Coulter, Brea, CA). The inflammatory markers procalcitonin (PCT), C-reactive protein (CRP), interleukin 6 (IL-6), D-dimer (DD), lactate dehydrogenase (LDH), and ferritin were done at baseline and at the time of discharge. Serum IL-6 concentration was estimated by using ELISA with a commercially available human IL-6 ELISA kit (Biotech Investissement, Besançon, France) as per the manufacturer's instructions. Serum ferritin, PCT, and D-dimer were measured by fluorescent immunoassay by sandwich immuno-detection method using i-Chroma analyzer [10].

The principal investigator, residents, doctors, and nursing staff were also trained to monitor and record in a datasheet after the asana-Pranayama schedule by direct observation during 7-10 days of hospital admissions. Clinical improvement/time to symptom abatement, total duration of hospital stay, and clinical outcomes of discharges/deaths were recorded. Vitals signs of oxygen saturation, body temperature, blood pressure, pulse rate, and respiratory rate were recorded as per protocol.

Statistical analysis

Data were analyzed using a Microsoft Office 2013 Excel worksheet. Categorical variables are presented as frequencies (%), and continuous data as mean ± SD. The student's t-test was performed, and a p-value less than 0.05 was considered statistically significant. MedCalc's Free statistical calculators were used for calculations: https://www.medcalc.org › calc.

## Results

**Figure 1:**
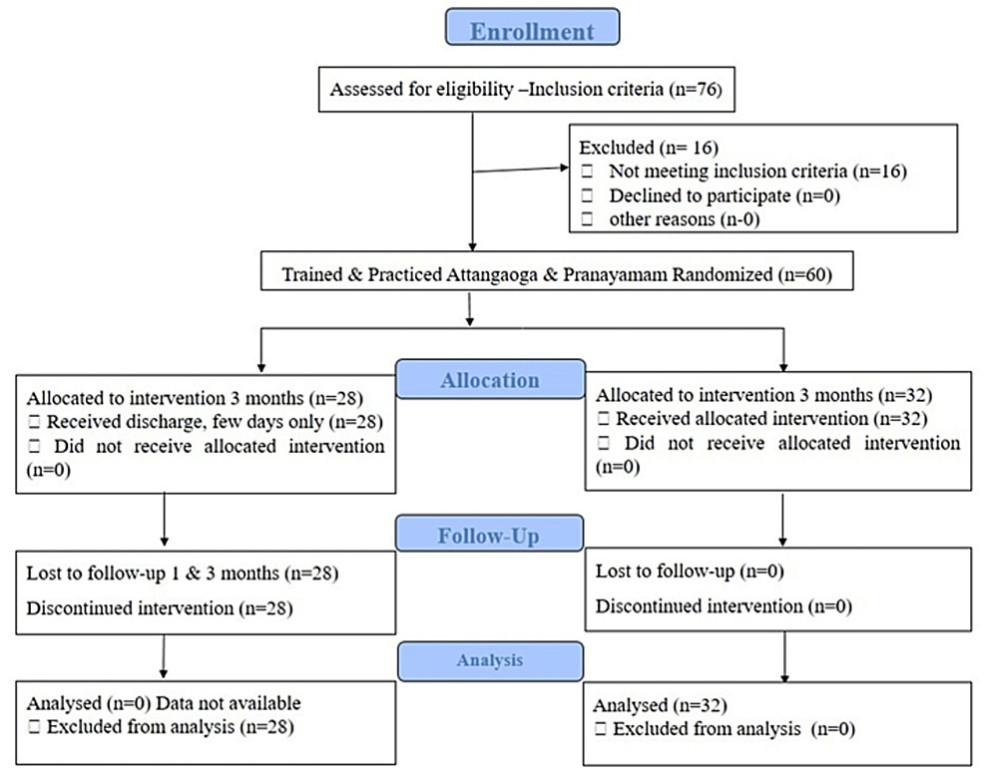
Flow diagram depicting the selection of study subjects

In the present study, 76 patients volunteered to participate and of them, 60 were trained; 32 patients regularly practiced Attangaogam (Athanam) yoga asana-Pranayamam as per the study protocol. A flow chart diagram of the recruitment process of the participants is presented in Figure 1. The patients were examined for eligibility; the data of patients who were confirmed eligible participated in the study and completed follow-ups were collected for final statistical analysis.

Of note, 73.2% of the study subjects were aged greater than 40 years. A CO-RADS score >3 was observed in 85% of the subjects. None of the patients had practiced yoga/Pranayamam before; only one patient had learned it but was not currently practicing it (Table 2). 

**Table 2:** Baseline characteristics of COVID-19-infected patients SD: standard deviation; MoHFW: Ministry of Health and Family Welfare; HFNC: high-flow nasal cannula; NIV: non-invasive ventilation; ICU: intensive care unit; CO-RADS: COVID-19 Reporting and Data System

Parameters	N (%)	Mean ± SD, range
Age group, years		
<20	1 (1.7%)	50.6 ± 4.95, 18–66
20-40	15 (25%)	
40-60	25 (42%)	
>60	19 (32%)	
Sex		
Male	37 (62%)	
Female	23 (38%)	
Disease severity on admission (MoHFW, India)		
Mild	13 (22%)	
Moderate	37 (62%)	
Severe	10 (17%)	
Oxygen saturation on admission		
<94%	35 (58%)	65–97
≥94%	25 (42%)	
Supplementation of oxygen on admission		
No oxygen supplementation (room air)	37 (62%)	0–8 L
Nasal cannula (conventional)	22 (36%)	
HFNC	1 (2%)	
NIV/ICU intubation	0	
CO-RADS score		
≤3	9 (15%)	4.08 ± 2, 1–6
>3	51 (85%)	
Alcohol consumption	2 (3%)	
Smoker	13 (22%)	
Yoga/Pranayama practice	1 (2%), currently not practicing	
COVID-19 vaccination status	1 (2%)	
Outcome: discharged; duration of stay in the hospital, days	60 (100%)	7.46 ± 3, 5–14

COVID-19 diagnostic inflammatory markers were found to be elevated in 17-98% of patients. Clinically, 82% of patients had a fever and dyspnoea, and 97% had myalgia. Low hemoglobin levels (<12 g/dl) were observed in 22% of patients, with a range of 7.6-15.5 g/dl (Table 3).

**Table 3:** Distribution of clinical characteristics and biochemical, hematological, and inflammatory markers on admission day among COVID-19-infected patients

Variables	Elevated reference value cut-off	N (%)	Range
Clinical presentations			
Fever, shortness of breath, dyspnea		49 (82%)	
Cough		37 (62%)	
Myalgia		58 (97%)	
Sore throat		4 (7%)	
Loss of smell/taste		12 (20%)	
Diarrhea		4 (17%)	
Comorbidity			
Hypertension		30 (22%)	
Diabetes mellitus		20 (32%)	
Others (renal disorders, asthma)		1 (1.7%)	
Medications			
Antivirals and steroids		39 (65%)	
Antibiotics		60 (100%)	
C-reactive protein (CRP)	CRP >10 mg/dl	50 (83%)	0.6–120
Procalcitonin	Normal (<0.1 ng/ml)	16 (27%)	0.09–10.16
	0.1-0.5, possible infection	43 (72%)	
	>2, severe sepsis	1 (2%)	
Fibrinogen	>500 mg/dl	10 (17%)	325–797
D-dimer	>500 ng/ml	18 (30%)	97–13601
Interleukin-6	>6 pg/ml	51 (85%)	1.3–240.09
Lactate dehydrogenase	>100 IU/L	59 (98%)	88–1716
Lymphocytes count	<20 x 10^9^/L	33 (55%)	3.9–50.9
Hemoglobin	<12 g/dl	30 (22%)	7.6–15.5

Post Attangaoga-Pranayamam practices, patients' oxygen saturation and respiratory rates attained normal levels (mean of 20.2 ± 2.4/minute on discharge day). All inflammatory markers were found to have decreased on discharge day and showed continued fall to attain normal limits at one- and three-month follow-ups (p<0.05) (Table 4).

**Table 4:** Comparison of clinical characteristics and inflammatory markers between pre- (admission day) and post-Attangaoga-Pranayama practice sessions among COVID-19-infected patients *Statistically significant (p<0.05) SD: standard deviation

Parameter	Admission day, mean ± SD	Discharge day, mean ± SD	Post 1 month, mean ± SD	Post 3 months, mean ± SD	Pre-post discharge p-value	Pre-post 1 month p-value	Pre-post 3 months p-value
Respiratory rate (12-16 breaths/minute)	24.2 ± 5.83	20.2 ± 2.4	17.4 ± 2.1	16.5 ± 1.3	0.0008*	0.0001*	0.0001*
Oxygen saturation >94-98%	84.2 ± 10	94.5 ± 2.5	98.5 ± 0.6	99 ± 0.5	0.0001*	0.0001*	0.0001*
C-reactive protein (up to 10 mg/dl)	23.1 ± 7.21	12.8 ± 5.2	4.7 ± 1.9	1.4 ± 0.5	0.0001*	0.0001*	0.0001*
Lactate dehydrogenase (60-100 IU/L)	802 ± 89.09	408 ± 79	156 ± 69	87 ± 43	0.0001*	0.0001*	0.0001*
Ferritin (ng/ml), male: 20-220	311 ± 282.06	288 ± 195	246 ± 104	196 ± 52	0.71	0.23	0.02*
Ferritin (ng/ml), female: 15-120	298 ± 187	143 ± 39	109 ± 55	69 ± 23	0.0001*	0.0001*	0.0001*
Fibrinogen (200-400 mg/dl)	361.0 ± 120.92	301 ± 39	-	-	0.0001*	-	-
Procalcitonin (<0.1 ng/ml)	0.34 ± 0	0.15 ± 0	0.1 ± 0	-	0.0001*	0.0001*	0.0001*
D-dimer (<500 ng/ml)	938 ± 202.94	719 ± 129	662 ± 106	306 ± 58	0.0001*	0.0001*	0.0001*
Interleukin-6 (5.3-7.5 pg/ml)	43.6 ± 4.03	29 ± 4.23	19 ± 1.2	-	0.0001*	0.0001*	-

**Figure 2:**
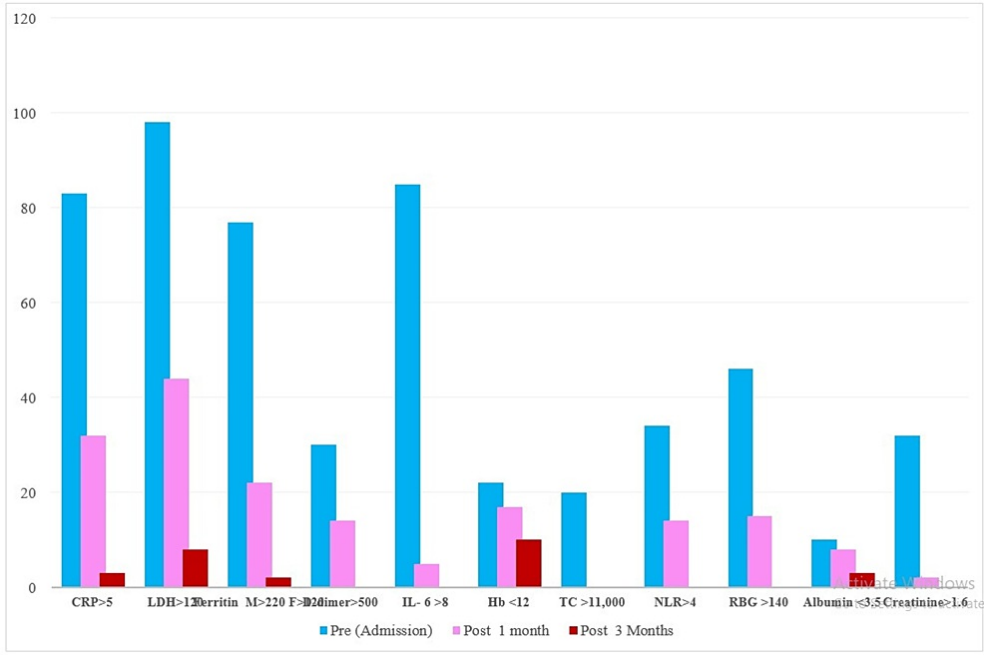
Percentage (%) of out-of-range blood investigations pre- (admission day) and post-Attangaoga-Pranayama practice sessions among COVID-19-infected patients CRP: C-reactive protein; LDH: lactate dehydrogenase; IL-6: interleukin 6; Hb: hemoglobin; TC: total leukocyte count; NLR: neutrophil-lymphocyte ratio; RBG: random blood glucose

The routine biochemical and hematological parameters improved after yoga-Pranayamam practices and were within normal limits by three months (Table 5, Figure 2).

**Table 5:** Comparison of hematological and routine biochemical investigations between pre- (admission day) and post-Attangaoga-Pranayama practice sessions among COVID-19-infected patients *Statistically significant (p<0.05) SD: standard deviation

Parameter	Admission day, mean ± SD	Discharge day, mean ± SD	Post 1 month, mean ± SD	Post 3 months, mean ± SD
Hemoglobin (g/dl)	12.8 ± 0.35	12.5 ± 0.5	12.3 ± 0.4	13.2 ± 0.3^*^
Total leukocyte count (x 10^9^/L)	8303 ± 3986	7459 ± 1698^*^	6287 ± 599^*^	6049 ± 789^*^
Neutrophil-lymphocyte ratio	8.85 ± 1.35	3.8 ± 1.5^*^	2.8 ± 1.2^*^	2.2 ± 1.3^*^
Random glucose (mg/dl)	189 ± 74	169 ± 82^*^	121 ± 25^*^	119 ± 16^*^
Serum albumin (g/dl)	3.9 ± 1.6	3.8 ± 1.5	3.8 ± 0.6	4.1 ± 0.5
Serum creatinine (mg/dl)	2.5 ± 1.3	1.8 ± 0.5^*^	1.1 ± 0.5^*^	1.0 ± 0.5^*^

## Discussion

General, clinical characteristics and oxygen saturation

This study reports the successful adjuvant treatment with yoga asana and Pranayamam practices among COVID-19 patients during the second wave in India. In this study, none of the participants reported worsening of any major symptoms of COVID-19 or any adverse effects during the follow-up. Attangaoga-Pranayamam practices helped patients to attain normal levels of oxygen saturation and respiratory rate (mean of 20.2 ± 2.4/minute on discharge day).

The mean age of the study population was 50.6 ± 4.95 years and 62% were males, which is similar to other studies. With regard to comorbidities, the prevalence of hypertension and diabetes mellitus was 22% and 32% respectively [11]. Smokers and alcoholics represented 22% and 3% of the cohort respectively. All patients experienced relief from COVID-19 symptoms on the third day of admission after taking part in Attangaoga-Pranayamam practice sessions. Cold, cough and respiratory symptoms disappeared on the fifth-sixth day of practice, and patients attained normal oxygen saturation and were discharged. A similar promising observation was found in other published case reports [12].

Of note, 58% of the patients had hypoxemia (sO_2 _<94%, range: 65-97%) and mean tachypnea of 24.2 ± 5.83 breaths/minute on admission. The mean CO-RADS score observed was 4.08 ± 2 and 85% of the patients had CO-RAD scores >3; 36% of these patients required intermittent conventional oxygen supplementation of 1-6 L of oxygen. However, none of the patients on yoga therapy required a high-flow nasal cannula (HFNC) or ICU transfer. While the duration of hospital stay among the patients in this study was low (mean: 7.46 ± 3 days, range: 5-14 days), the study by Kapoor et al. showed that supplementation of oxygen itself was required for 8.1 ± 3.1 days [9]. Antiviral therapy and steroids were therapeutically given to 65% of patients [9]. The easy recovery observed in these patients was due to the regularity of performing all Pranayamam sessions, which has been recommended by other studies as well [1,13]. In addition, the Attangaoga practices with the help of multiple poses, breathing, and relaxation techniques proved helpful in strengthening the inspiratory and expiratory muscles and reducing dyspnea, enhancing the strength of the lungs, improving oxygenation and pleural pressure gradient, and increasing lung volume with alveolar ventilation, and facilitated the drainage of secretions as discussed in other studies on yoga in the literature [8,14].

All patients attained normal oxygen saturation and became asymptomatic within a week, and were discharged after mean stays of 7.8 ± 0.71, 8.3 ± 0.71, and 8.9 ± 4.9 days from the hospital for mild, moderate, and severe categories respectively. In this study, those patients aged more than 50 years, and those (17%) in the severe category with comorbidities of diabetes mellitus and hypertension who practiced Attangaoga-Pranayamam also had an early recovery compared to other admitted patients of similar age and gender who did not practice Attangaoga-Pranayamam. Although they had comparatively longer stays in the hospital, there were no complications during their home-quarantine period. Similar promising results were observed in the case report by Mishra et al. [12].

Biochemical, inflammatory, and hematological markers

The blood investigations of study subjects revealed the elevation of various inflammatory markers, as shown in Table 3. These findings are comparable to previous reports documenting COVID-19 infections on admission [10,11]. The data on elevated levels of various inflammatory markers such as CRP >10 mg/dL (83%), LDH >100 IU/L (98%), IL-6 >6 pg/ml (85%), ferritin (males >220 ng/ml, females: >120 ng/ml) (77%), D-dimer >500 ng/ml (30%), and PCT >0.1 ng/ml (74%) at the time of admission is shown in Figure 2.

The comparison of clinical, biochemical, and inflammatory parameters between pre- (admission day) and post-Attangaoga-Pranayama practice sessions on the discharge day and post-one and three months showed a decreasing trend and patients attained normal levels by three months; the difference was statistically significant (p<0.05), as depicted in Table 3.

CRP is a molecule synthesized by the liver, which senses the balance between pro- and anti-inﬂammatory cytokines. The mean CRP value on admission among the participants in this study was 23.1 mg/dl; post-asana-Pranayamam, on discharge day, and at one month, and three months, the mean CRP values observed were 12.5 mg/dl, 4.7 mg/dl, and 1.4 mg/dl respectively. IL-6 is a pleiotropic cytokine, secreted by cells of innate and adaptive immune systems as a response to microbial antigens. It induces the secretion of CRP, which helps in the activation of the classical complement pathway, thereby facilitating the mediation of phagocytosis. IL-6 has been proposed to be a good marker of prognosis and contributes to effective host defense against COVID-19 infection. In our study, the baseline IL-6 levels of 43.6 ± 4.03 pg/ml decreased gradually and the difference was statistically significant. The beneficial effect of Pranayamam prevented the cytokine storm of extensive production of IL-6, thereby inhibiting the severe systemic inflammatory response. So, none of the study patients required tocilizumab IL-6 blockade therapy, which resulted in less economic burden [8,15]. PCT makes a better distinction between bacterial infection and other inflammatory processes than TLC or CRP levels. The mean PCT value never reached severe sepsis levels (PCT >2 ng/ml) in our study as estimated on the discharge day (0.15 ± 0) [16].

The mean D-dimer levels in the patients were 934 ± 202.9 ng/ml on admission and reached normal levels by three months, and hence there were no incidences of hypercoagulation or complications of disseminated intravascular coagulopathy (DIC). None of the patients experienced bacterial sepsis and DIC as inflammatory status was controlled by performing asana-Pranayamam on a daily basis. LDH value ranged from 88- 1716 IU/L with a mean of 802 ± 89 IU/L on admission. LDH is an enzyme that helps in the interconversion of lactate to pyruvate in the cells, an immune surveillance prognostic biomarker [10,13]. The Attangaogam benefitted patients who practiced it and only 8% of them showed LDH elevation after practice, whereas in another study, 27% of patients had continued LDH elevation due to persistent inflammation [17].

Of note, 77% of the patients showed ferritin elevation at the time of admission, evidencing immune dysregulation and indicating life-threatening hyperinflammation because of cell damage [10], which decreased to 22% post-one month and to 3% post-three months. The mean hemoglobin was 12.8 ± 0.35 g/dl, and TLC was 8303 ± 3986, which is comparable to other published studies [16-18]. The persistence of LDH >120 IU/L and Hb <12 g/dl in 8-10% of patients may have been due to additional nutritional requirements for the correction of anemia during convalescence. The mean serum albumin was 3.9 g/dl on admission, 3.7 g/dl on discharge, and 3.8 g/dl post-one month, which improved to 4.1 g/dl post-three months. The virus-activated inﬂammation of cellular stress promoting a hypercatabolic/hypermetabolic state was conﬁrmed in the patients as evidenced by a reduction in circulating albumin and its gradual attainment of normal reference levels [17]. The hematological parameters indicating systematic inflammatory response, like TLC and neutrophil-lymphocyte ratio (NLR) [16], continued to decrease and attained normal levels by one month.

The patients' hyperglycemic status was brought under control with the aid of Attangaoga-Pranayamam practices within one month, as shown in Table 4. The mean random blood glucose on admission was 189 ± 74 mg/dl, while that post-one month was 121 ± 25 mg/dl, and the difference was statistically significant (p = 0.001) [9]. Many patients did not require steroids and others were able to discontinue steroids on hospital discharge. Serum creatinine showed a mild elevation at 2.5 ± 1.3 mg/dl on admission, which returned to normal levels by one month. Yoga and Pranayamam have been shown to down-regulate the hypothalamic-pituitary-adrenal axis by reducing perceived stress and inflammation and improving respiration, the sense of well-being, and immune functions [18]. Hence, COVID-19-infected patients recovered and became fully healthy by practicing 21 asanas and nine Pranayamam protocols once daily.

Limitations and recommendations

This study has a few limitations, which are as follows: (1) no assessments of pulmonary function test or antibody titer were performed and (2) we could not include a control group as subjects regularly practicing yoga asanas (Athanam)-Pranayamam were not infected, and if infected, they had recovered with home isolation. No hospitalization with special investigations and supplementation of oxygen was required. Future studies adopting a randomized controlled trial design with larger sample sizes are required to gain more insights into adopting this treatment strategy in all COVID-19 patients.

## Conclusions

The study reported the successful adjuvant treatment of COVID-19 patients with several comorbidities by employing Attangaoga-Pranayamam practices. The Pranayamam practices help in restoring respiratory system functions and prevent the further lowering of oxygen saturation. The evidence related to biomarkers revealed that the patients attained metabolic normalcy of cell health with yoga rehabilitation therapy by counteracting inﬂammation and promoting tissue repair and natural innate immunity. This research endorses the benefits of yoga and Pranayamam as a holistic treatment method for general human well-being and health.
